# Visibilidade e voz para as vítimas de violência doméstica no Brasil

**DOI:** 10.1590/0102-311XPT202025

**Published:** 2026-02-16

**Authors:** Bruna Lana Camargos Costa, Adalgisa Peixoto Ribeiro

**Affiliations:** 1 Universidade Federal de Minas Gerais, Belo Horizonte, Brasil.

A violência contra as mulheres, em termos conceituais, se inscreve no bojo do conceito de violência de gênero, que se caracteriza por qualquer ato, seja físico, psicológico ou sexual, cometido contra outra pessoa devido ao seu gênero. Emerge nas relações hierárquicas de poder, em que a pessoa subalterna não é considerada igual ou com as mesmas condições de valor e existência ^1^. Nesse sentido, a violência ocorre para manter a desigualdade de poder. É importante destacar que a violência de gênero não atinge somente as mulheres, mas as têm como seu alvo mais frequente ^2^.

Grande parte das violências contra as mulheres ocorre em ambiente familiar ou doméstico com consequências que se estendem não só para a vítima direta, mas também para quem está em seu entorno. Para as mulheres vítimas, essas consequências podem incluir desde problemas emocionais, incapacidades temporárias ou permanentes, até a morte. As repercussões da violência contra as mulheres, quando é perpetrada pelo parceiro íntimo atual ou anterior atinge também a família, principalmente os filhos.

A discussão sobre o tema, assim como sua visibilidade nos mais diversos âmbitos da vida em sociedade, seja na mídia ou nos espaços acadêmicos e científicos, tem ganhado espaço. No entanto, sua magnitude ainda não está totalmente desvelada. Dados de registros oficiais do sistema de saúde brasileiro mostram que, de 2013 a 2023, mais de 47 mil mulheres foram assassinadas, o que equivale a 13 mortes por dia. Somente em 2023, foram 3.903 mulheres mortas de forma violenta, com uma taxa de 3,5 mulheres por 100 mil habitantes do sexo feminino, taxa que permaneceu inalterada entre 2022 e 2023, enquanto se registrou um decréscimo de 2,3% na taxa geral de homicídios ^3^.

É nesse contexto que Ana Paula Araújo, jornalista de reconhecida trajetória na cobertura de temas silenciados, reafirma seu compromisso com a denúncia e a conscientização da sociedade ao lançar o livro *Agressão: A Escalada da Violência Doméstica no Brasil* ^4^. Esta é a segunda obra da autora sobre violência contra mulheres. Se em seu primeiro livro *Abuso: A Cultura do Estupro no Brasil* ^5^ a autora expõe como o crime da violência sexual é socialmente naturalizado, neste novo trabalho ela volta seu olhar para a violência praticada por parceiros íntimos. Trata-se de uma ameaça à vida das mulheres, muitas vezes encoberta pelo afeto, dependência emocional, medo e vergonha. O livro está estruturado em 15 capítulos, que reúnem entrevistas com mulheres de diferentes perfis, classes sociais, idades e etnias. A autora percorreu todas as regiões do país durante quatro anos e, além das mulheres vítimas, conversou também com agressores, familiares, profissionais de saúde e representantes do Judiciário.

Na introdução, a autora compartilha sua motivação pessoal e profissional para retornar ao tema da violência contra mulheres, agora com foco na violência doméstica. Reafirma que o livro nasceu da necessidade de compreender os mecanismos que perpetuam essa violência, mesmo diante da existência de uma das legislações mais avançadas do mundo no enfrentamento à violência contra a mulher, a Lei Maria da Penha, em vigor no Brasil desde 2006 e com alterações mais recentes que a tornaram ainda mais dura com os agressores e mais protetiva em relação às vítimas.

Embora a obra apresente uma abordagem jornalística, sua narrativa levanta questões importantes para a saúde pública. O trabalho da autora ajuda a compreender as múltiplas formas em que a violência se expressa, suas repercussões na vida e na saúde das vítimas, sejam elas diretas ou indiretas. Além disso, seu discurso destaca a necessidade urgente de políticas públicas mais eficazes, de qualificar o debate público e implementar estratégias de prevenção primária. Essas estratégias devem integrar de forma articulada o sistema de saúde e os diferentes setores da sociedade para proteger e acolher as vítimas.

No conjunto de relatos, observa-se a recorrência de elementos que revelam padrões estruturais da violência de gênero no contexto doméstico. Um dos pontos comuns nas entrevistas é o medo de retaliação por parte dos agressores, além de sentimentos de vergonha e culpa, que têm impacto significativo no subregistro de denúncias. Além disso, é evidente que a maioria das agressões acontece em casa. Outro ponto comum é o sofrimento dos filhos, que é frequentemente citado como fator decisivo para que as mulheres busquem ajuda e façam denúncias formais. Ao mesmo tempo, diversas vítimas relatam a falta de escuta qualificada e a revitimização em instituições como delegacias, serviços de saúde e assistência social. Outras pesquisas apontam que, ao tentar buscar apoio, as vítimas ainda enfrentam barreiras de ordem estrutural, emocional e institucional, o que as torna ainda mais vulneráveis ^6^. A revitimização ou vitimização secundária, indicada pela autora do livro, ocorre quando a vítima, ao buscar ajuda, é submetida a experiências que a fazem reviver o trauma já sofrido. Isso pode acontecer em diferentes momentos após o evento violento, como no caso de vítimas de abuso sexual que precisam relatar repetidas vezes os detalhes da agressão a profissionais em diversos serviços, prolongando e intensificando o sofrimento ^7^.

Em quase todas as entrevistas retratadas no livro, a violência começou de maneira sutil, com abusos psicológicos, que se transformaram gradualmente em formas mais evidentes e até mesmo letais. A maior parte das vítimas relata o ciclo tradicional da violência, que inclui as fases de tensão, agressão e reconciliação. Tal dinâmica foi sistematizada pela psicóloga estadunidense Lenore Walker que, ao investigar os padrões recorrentes em relacionamentos abusivos, formulou a Teoria do Ciclo da Violência, com três fases: na primeira, o agressor apresenta tensão crescente, com irritação, ameaças e ofensas; na segunda, a violência se concretiza; e na terceira, chamada de reconciliação ou “lua de mel”, surge o aparente arrependimento, quando o agressor adota postura conciliadora, levando a vítima a acreditar na possibilidade de mudança e no fim das agressões ^8^.

Por outro lado, embora haja elementos comuns, os relatos também apresentam diferenças que exigem análise mais cuidadosa. As experiências e reações das entrevistadas à violência são afetadas diretamente por marcadores sociais como classe e nível de escolaridade, além de origem geográfica e contexto cultural. Essas variáveis influenciam tanto a diversidade de estratégias de enfrentamento utilizadas, quanto os diferentes níveis de acesso aos serviços de proteção. Além disso, surgem formas contemporâneas de violência, como a exposição da privacidade online, que intensificam os métodos de controle e humilhação empregados pelos agressores. Embora essas diferenças não invalidem os padrões estruturais identificados, elas destacam a importância de abordagens interseccionais que levem em conta as particularidades de cada trajetória, a fim de promover respostas mais eficazes e sensíveis às diversas facetas da violência doméstica.

O trabalho da autora evidencia que a violência doméstica compromete de forma significativa a saúde física, emocional e social das vítimas, ao mesmo tempo em que impõe uma sobrecarga expressiva ao sistema público de saúde. Como resultado das agressões, nota-se a necessidade de atendimentos recorrentes nos serviços de saúde dos diversos níveis (desde a atenção básica até unidades hospitalares) e cuidados prolongados, necessários para o tratamento de lesões físicas e transtornos emocionais. Ao final do livro, a autora traz uma lista de instituições às quais as mulheres poderão se dirigir em busca de apoio e, se dirigindo ao público leigo, lembra que qualquer pessoa que tenha conhecimento pode denunciar a violência.

A partir das entrevistas realizadas, o livro de Ana Paula Araújo tem elementos suficientes para uma reflexão mais aprofundada e para uma discussão com a literatura de outros campos do conhecimento, o que não foi realizado pela autora. No entanto, cumpre seu objetivo e papel de ampliar a visibilidade das histórias e experiências das vítimas diretas e indiretas da violência doméstica contra as mulheres de diversas classes sociais, faixas etárias, visibilidades e invisibilidades e de distintas regiões do país. Neste sentido, amplia o debate sobre suas repercussões na sociedade e chama a todos para a reflexão sobre esta importante ameaça à vida das mulheres, mesmo em contexto de avanços na legislação que pretende protegê-las.
